# Early cardiac changes in a rat model of prediabetes: brain natriuretic peptide overexpression seems to be the best marker

**DOI:** 10.1186/1475-2840-12-44

**Published:** 2013-03-07

**Authors:** Sara Nunes, Edna Soares, João Fernandes, Sofia Viana, Eugénia Carvalho, Frederico C Pereira, Flávio Reis

**Affiliations:** 1 Laboratory of Pharmacology and Experimental Therapeutics, IBILI, Faculty of Medicine, University of Coimbra, Coimbra 3000-548, Portugal; 2 Biochemistry Department, Faculty of Pharmacy, University of Porto, Porto, Portugal; 3 Institute for Molecular and Cellular Biology, University of Porto, Porto, Portugal; 4 Center for Neuroscience and Cell Biology, University of Coimbra, Coimbra, Portugal; 5 The Portuguese Diabetes Association (APDP), Lisbon, Portugal

**Keywords:** Brain natriuretic peptide, Diabetic cardiomyopathy, Fibrosis, Hypertrophy, High-sucrose diet, Prediabetes

## Abstract

**Background:**

Diabetic cardiomyopathy (DCM) is defined as structural and functional changes in the myocardium due to metabolic and cellular abnormalities induced by diabetes mellitus (DM). The impact of prediabetic conditions on the cardiac tissue remains to be elucidated. The goal of this study was to elucidate whether cardiac dysfunction is already present in a state of prediabetes, in the presence of insulin resistance, and to unravel the underlying mechanisms, in a rat model without obesity and hypertension as confounding factors.

**Methods:**

Two groups of 16-week-old Wistar rats were tested during a 9 week protocol: high sucrose (HSu) diet group (n = 7) – rats receiving 35% of sucrose in drinking water vs the vehicle control group (n = 7). The animal model was characterized in terms of body weight (BW) and the glycemic, insulinemic and lipidic profiles. The following parameters were assessed to evaluate possible early cardiac alterations and underlying mechanisms: blood pressure, heart rate, heart and left ventricle (LV) trophism indexes, as well as the serum and tissue protein and/or the mRNA expression of markers for fibrosis, hypertrophy, proliferation, apoptosis, angiogenesis, endothelial function, inflammation and oxidative stress.

**Results:**

The HSu-treated rats presented normal fasting plasma glucose (FPG) but impaired glucose tolerance (IGT), accompanied by hyperinsulinemia and insulin resistance (P < 0.01), confirming this rat model as prediabetic. Furthermore, although hypertriglyceridemia (P < 0.05) was observed, obesity and hypertension were absent. Regarding the impact of the HSu diet on the cardiac tissue, our results indicated that 9 weeks of treatment might be associated with initial cardiac changes, as suggested by the increased LV weight/BW ratio (P < 0.01) and a remarkable brain natriuretic peptide (BNP) mRNA overexpression (P < 0.01), together with a marked trend for an upregulation of other important mediators of fibrosis, hypertrophy, angiogenesis and endothelial lesions, as well as oxidative stress. The inflammatory and apoptotic markers measured were unchanged.

**Conclusions:**

This animal model of prediabetes/insulin resistance could be an important tool to evaluate the early cardiac impact of dysmetabolism (hyperinsulinemia and impaired glucose tolerance with fasting normoglycemia), without confounding factors such as obesity and hypertension. Left ventricle hypertrophy is already present and brain natriuretic peptide seems to be the best early marker for this condition.

## Background

Diabetic cardiomyopathy (DCM) is defined as structural and functional changes in the myocardium, independent of hypertension, chronic artery disease (CAD) or any other known cardiac diseases, which are caused by metabolic and cellular abnormalities induced by diabetes mellitus (DM), ultimately resulting in heart failure (HF) [1-3]. Cardiovascular diseases (CVD) are responsible for three quarters of the deaths among the diabetic population [4] and diabetes accounts for a significant percentage of patients with a diagnosis of HF, which is a major cause of mortality and morbidity in these patients [5,6].

The transition from the early metabolic abnormalities that precedes diabetes, for example impaired fasting glucose (IFG) and impaired glucose tolerance (IGT), to diabetes may take many years; however, current estimates indicate that most individuals with these pre-diabetic states eventually develop DM [7-9]. DCM may be subclinical for a long time, before the appearance of clinical symptoms or signs, complicating its diagnosis and aggravating future complications. However, during the prediabetic state, the risk of cardiovascular events is already increased and myocardial abnormalities might appear prior to the diagnosis of type 2 DM. Thus, the earlier identification of cardiac changes in prediabetic/insulin resistant patients could be a better strategy to prevent the evolution to most serious stages of the disease.

One of the most important structural hallmarks of DCM is cardiac hypertrophy [10,11], and this, in turn, is a powerful predictor of CV events [12]. Hyperglycemia has been viewed as the pivotal pathogenetic factor for the development of DCM. In fact, it can cause abnormalities at the cardiac myocyte level, eventually leading to functional and structural abnormalities, including systolic and diastolic dysfunction, as well as cardiac hypertrophy and myocardial fibrosis [13]. However, other factors seem to be involved in the evolution of the disease, including hyperinsulinemia, insulin resistance, oxidative stress, inflammation, endothelial dysfunction and apoptosis [10,11,14]. During the last years, the pathological, structural, functional and molecular aspects of the disease have been increasingly investigated, but the issue is far to being elucidated. The knowledge of the cellular and molecular aspects underlying the metabolic disturbances on cardiomyocytes in the prediabetic state should be useful in predicting the structural and functional cardiac consequences. Animal models have been used to study the mechanisms underlying DCM [15-18], but there are limitations. Indeed, the presence of important confounding factors displaying the susceptibility to cardiomyopathy, such as obesity and hypertension, as well as the scarce animal models of prediabetes are major impediments in the progress and understanding of the disease.

Thus, the purpose of this work was to elucidate whether cardiac alteration are already present in a pre diabetic state, and to study its underlying mechanisms, using the high-sucrose (HSu) diet rat model, which is associated with minor metabolic abnormalities and might mimic the human prediabetic state of insulin resistance [19,20], without other complicating factors that could lead to cardiac events.

## Material and methods

### Animals and diet

Male adult (16 weeks-old) Wistar rats (Charles River Laboratories, Barcelona, Spain), weighting 332.9 ± 9.0 g were maintained under controlled temperature (22–23°C) and light (12:12-h light–dark cycle). After 1 week of acclimatization, animals were randomly divided into two groups (n = 7 each), for a 9-weeks protocol: control – rats continued to receive tap water for drinking; and high-sucrose diet (HSu), rats received 35% sucrose (S0389; Sigma-Aldrich) in the drinking water. All animals were fed standard rat chow, containing 16.1% of protein, 3.1% of lipids, 3.9% of fibers and 5.1% of minerals (AO4 Panlab, Barcelona, Spain) *ad libitum* (with exception in the fasting periods). Food and beverage consumption was monitored for both groups throughout the experiment. All experiments were conducted according to the National and European Directives on Animal Care, as well as with ARRIVE guidelines on animal research [21]. The body weight (BW) of each animal was recorded weekly during the experimental period, using an analytical balance (KERN CB 6 K1, Germany).

### Blood pressure and heart rate assessment

Systolic (SBP), diastolic (DBP), mean blood pressure (MBP) and heart rate (HR) values were evaluated in conscious rats using a tail-cuff sphygmomanometer LE 5001 (Letica, Barcelona, Spain) in appropriate contention cages.

### Blood and tissue collection and preparation

At the final time, the rats were subjected to intraperitoneal anesthesia with 50 mg/kg pentobarbital (Sigma-Aldrich, Portugal) solution and a blood sample was immediately collected by venipuncture from the jugular vein into syringes with Heparin-Lithium (Sarstedt, Monovette®) for plasma samples and into needles without anticoagulant (for serum samples). The rats were then sacrificed by cervical dislocation, and the heart was immediately removed, placed in ice-cold Krebs’ buffer, carefully cleaned of adherent fat and connective tissue, weighted and divided in left and right ventricle. Heart regions were frozen in liquid nitrogen and stored at −80°C, for biochemical or gene expression analysis. Before sampling, heart weight (HW) and left ventricle weight (LVW) were measured in order to be used as cardiac trophism indexes in relation to body weight: HW/BW and LVW/BW.

### Metabolic characterization

Glucose tolerance test (GTT) was performed in fasted rats (6-h) injected intraperitoneally (i.p.) with a glucose bolus of 2 g/kg BW. The tail vein blood glucose levels were measured using a portable device (One Touch UltraEasy® glucometer, Lifescan, Johnson and Johnson, Portugal) in samples taken immediately before the bolus and 15, 30, 60, and 120 minutes after. Glycemia was measured in fed conditions.

Insulin tolerance test (ITT) was performed after an i.p. injection of 0.75 U/kg BW of insulin (I9278, Sigma), in 6-h fasted rats, through monitoring the blood glucose before the injection and 15, 30, 45, 60 and 120 min. after, using the same glucometer. Fasting insulin levels were quantified by using a rat insulin ELISA kit (Mercodia, Uppsala, Sweden). Insulin sensitivity of individual animals was evaluated using the homeostasis model assessment (HOMA) index [22]. The formula used was as follows: [HOMA-IR] = fasting serum glucose (mg/dL) × fasting serum insulin (μU/mL)/22.5. The values used (insulin and glucose) were obtained after an overnight fasting period.

Serum total cholesterol (TC) and triglycerides (TGs) were analyzed by enzymatic methods using an automatic analyzer (Hitachi 717, Roche Diagnostics). Total-cholesterol reagents and TGs kits were obtained from bioMérieux (Lyon, France).

### Serum and heart muscle protein levels and redox status

Serum levels of transforming growth factor β-1 (TGF-β1), vascular endothelial growth factor (VEGF) and interleukin-6 (IL-6) were measured through micro-ELISA sandwich assay, using commercial ultrasensitive Quantikine® ELISA kits (R&D Systems, Minneapolis, USA).

Cardiac expression of the receptor for advanced glycation endproducts (RAGE) and of tumour necrosis factor alpha (TNF-α) were quantified by western blot. The extracts were obtained from the left ventricle and were homogenized in 1.5 ml of RIPA lysis buffer (150 mM NaCl; 50 mM Tris–HCl pH = 8.0; 5 mM EGTA; 1% Triton X-100; 0.5% DOC; 0.1% SDS), supplemented with a protease inhibitor cocktail (1 mM phenylmethylsulfonyl fluoride, 1 mM dithiothreitol, 1 μg/mL chymostatin, 1 μg/mL leupeptin, 1 μg/mL antipain and 5 μg/mL pepstatin A; Sigma-Aldrich) and centrifuged 3 times (15500 × g, 15 min., 4°C). The resulting supernatant fraction (corresponding to total extract) was collected and total protein concentration was determined using BCA assay [23], supernatants were stored at –80°C until further use. Known amounts of total protein (25 μg for RAGE and 90 μg for TNF-α) were loaded and separated by electrophoresis on sodium dodecyl sulfate polyacrylamide gel electrophoresis (7.5% and 10%, respectively), transferred to a 0.45 μm polyvinylidene difluoride (PVDF) membranes (Immobilon, Millipore, Madrid, Spain) and blocked with 1% bovine serum albumin (BSA) in phosphate buffer saline with 0.1% Tween-20 (PBS-T) for 1 h at room temperature. Membranes were then incubated overnight at 4°C with primary antibody (rabbit anti-RAGE, 1:1000; anti-TNF-α, 1:600) both from Abcam (Cambridge, UK). The membranes were washed extensively in 0.1% PBS-T and then incubated for 1 h at room temperature with alkaline phosphatase conjugated secondary antibodies (goat anti-rabbit, 1:5000, GE Healthcare). Finally, membranes were visualized on Typhoon FLA 900 (GE Healthcare Bio-sciences) imaging system, using an enhanced chemifluorescence detection reagent (ECF, GE Healthcare). To confirm equal protein loading and sample transfer, membranes were reprobed with β-actin (1:5,000; Sigma-Aldrich) antibodies. Densitometric analyses were performed using the Image Quant 5.0 software. Results were normalized against β-tubulin, and then expressed as percentage of control.

The thiobarbituric acid reactive-species (TBARs) assay was used to evaluate serum and heart muscle tissue lipid peroxidation, via malondialdehyde (MDA). Samples were analyzed spectrophotometrically at 532 nm using 1,1,3,3-tetramethoxypropane as an external standard. Serum total antioxidant status (TAS) was measured through ferric reducing antioxidant potential (FRAP) assay, as previously described [24].

### RT-qPCR cardiac muscle gene expression

The heart was isolated and stored in RNA later™ solution (Ambion, Austin, TX, USA). In brief, tissue lysates were processed according to the protocol from RNeasy® Mini Kit (Qiagen, Hilden, Germany). RNA integrity (RIN, RNA Integrity Number) was analyzed using 6000 Nano Chip® kit, in Agilent 2100 bioanalyzer (Agilent Technologies, Walbronn, Germany) and 2100 expert software, following manufacturer instructions. The yield from isolation was from 0.5 to 3 μg; RIN values were 6.0–9.0 and purity (A260/A280) was 1.8–2.0. *Reverse transcription and relative quantification of gene expression were* performed as previously described [25]. Real-time PCR reactions were performed using the following primer sequences for Bax, Bcl2, brain natriuretic peptide (BNP), connective tissue growth factor (CTGF), intercellular adhesion molecule 1 (ICAM-1), IL-6, inducible nitric oxide synthase (iNOS), Pro-collagen type III, superoxide dismutase (SOD), TGF-β1, TNF-α, thrombospondin 1 (TSP-1), tribbles 3 (TRB3), vascular cell adhesion molecule 1 (VCAM-1) and VEGF, which were normalized in relation to the expression of beta-actin as an internal control: Bax Forward: 5^′^-CCAAGAAGC TGAGCGAGTGTCTC-3^′^ and Bax Reverse: 5^′^-AGTTGC CATCAGCAAACATGTCA-3^′^; Bcl-2 Forward: 5^′^-GGAG CGTCAACAGGGAGATG-3^′^ and Bcl-2 Reverse: 5^′^-GA TGCCGGTTCAGGTACTCAG-3^′^; BNP Forward: 5^′^-G GGCTGTGACGGGCTGAGGTT-3^′^ and BNP Reverse: 5^′^-AGTTTGTGCTGGAAGTAAGA-3^′^; CTGF Forward: 5^′^-CGTAGACGGTAAAGCAATGG-3^′^ and CTGF Reverse: 5^′^-AGTCAAAGAAGCAGCAAACAC-3^′^; ICAM-1 Forward: 5^′^-TTCAACCCGTGCCAGGC-3^′^ and ICAM-1 Reverse: 5^′^-GTTCGTCTTTCATCCAGTTAGTCT-3^′^; IL-6 Forward: 5^′^-ACCACTTCACAAGTCGGAGG-3^′^ and IL-6 Reverse: 5^′^-ACAGTGCATCATCGCTGTTC-3^′^; iNOS Forward: 5^′^-CAGAAGCAGAATGTGACCATCAT-3^′^ and iNOS Reverse: 5^′^-CGGAGGGACCAGCCAAATC-3^′^; Pro-collagen type III Forward: 5^′^-5^′^-GGTCACTTTCACT GGTTGACGA-3^′^ and Pro-collagen type III Reverse: 5^′^-TTGAATATCAAACACGCAAGGC-3^′^; SOD Forward: 5^′^-GACAAACCTGAGCCCTAACGG-3^′^ and SOD Reverse: 5^′^-CTTCTTGCAAACTATG-3^′^; TGF-β1 Forward: 5^′^-ATACGCCTGAGTGGCTGTCT-3^′^ and TGF-β1 Reverse: 5^′^-TGGGACTGATCCCATTGATT-3^′^; TNF-α Forward: 5^′^-CACGCTTTCTGTCTACTGA-3^′^ and TNF-α Reverse 5^′^-GGACTCCGTGATGTCTAAGT-3^′^; TSP-1 Forward: 5^′^-CTTTGCTGGTGCCAAGTGTA-3^′^ and TSP-1 Reverse: 5^′^-CGACGTCTTTGCACTGGATA-3^′^; TRB3 Forward: 5^′^-TGATGCTGTCTGGATGACAA-3^′^ and TRB3 Reverse: 5^′^-GTGAATGGGGACTTTGGTCT-3^′^; VCAM-1 Forward: 5^′^-GAAGCCGGTCATGGTCAAGT-3^′^ and VCAM-1 Reverse: 5^′^-GACGGTCACCCTTGAACAG TTC-3^′^; VEGF Forward: 5^′^-GAGAATTCGGCCCCAA CCATGAACTTTCTGCT-3^′^ and VEGF Reverse: 5^′^-G AGCATGCCCTCCTGCCCGGCTCACCGC-3^′^; beta-actin Forward: 5^′^-TGTGCTATGTTGCCCTAGACTT C-3^′^ and beta-actin Reverse: 5^′^-CGGACTCATCGTA CTCCTGCT-3^′^. Results were analyzed with SDS 2.1 software (Applied Biosystems, Foster City, CA, USA) and relative quantification calculated using the 2 − ΔΔCt method [26].

### Statistical analysis

Results were expressed as means ± standard errors of the mean (S.E.M.) and % of the Control, as indicated. Comparisons between groups were analyzed by the unpaired Student’s *t*-test, using GraphPad Prism software, Version 5.0. Differences were considered to be significant at *P* <0.05.

## Results

### The HSu-diet as a rat model of prediabetes with impaired glucose tolerance and insulin resistance

**Figure 1:**
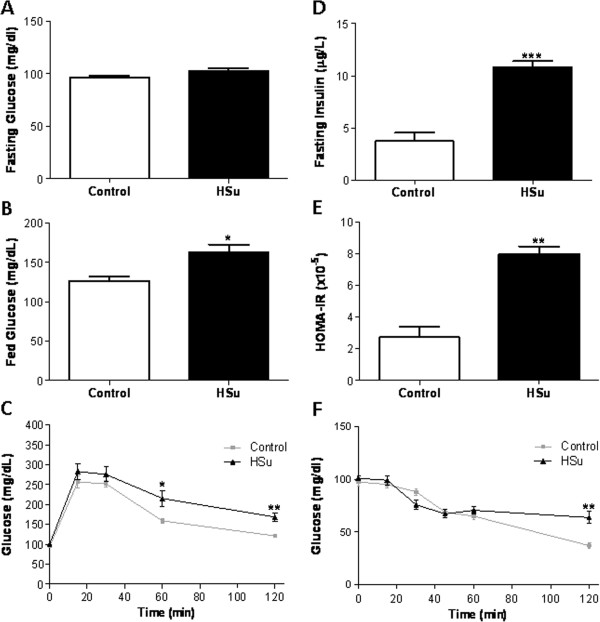
**Metabolic characterization of the prediabetic animal model.** Glycemic and insulinemic profile: fasting glucose (**A**), fed glucose (**B**), glucose tolerance test (**C**), fasting insulin (**D**), HOMA-IR index (**E**) and insulin tolerance test (**F**), after 9 weeks of treatment. (Results are mean ± SEM; n = 7 per group. *p < 0.05; **p < 0.01, ***p < 0.001 versus Control group).

Beverage consumption was unchanged between groups throughout the experiment. The HSu-treated rats showed a BW profile similar to that of the control animals, during the 9 weeks of treatment (Table 1). Similarly, fasting glycemia was unchanged between the two groups (102.90 ± 6.98 vs 96.71 ± 4.49 mg/dl) (Figure 1A); however, in the fed state, blood glucose levels were significantly elevated in the HSu group compared to Control (162.90 ± 26.54 *vs* 126.80 ± 13.56 mg/dL; p < 0.05) (Figure 1B). During a GTT, the HSu-treated rats showed significantly increased blood glucose levels when compared with those of the Control group (215.50 ± 19.64 *vs* 159.00 ± 5.83 mg/dl; p < 0.05), 60 min. after the injection of glucose. This difference persisted until the 120 min., when the blood glucose concentrations returned to basal levels in the Control group, but remained significantly elevated in the HSu-treated group (168.80 ± 10.64 *vs* 121.40 ± 3.69 mg/dL; p < 0.01) (Figure 1C).

**Table 1:** Body, heart and left ventricle weights, blood pressure, heart rate and lipid profile

**Parameter**	**Control group (n = 7)**	**HSu group (n = 7)**
*Body, heart and LV weights*		
BW (g)	390.40 ± 8.45	397.40 ± 8.71
HW (g)	0.89 ± 0.09	1.10 ± 0.01*
LVW (g)	0.45 ± 0.02	0.53 ± 0.02***
HW/BW (g/kg)	2.14 ± 0.23	2.62 ± 0.07
LVW/BW (g/kg)	1.07 ± 0.02	1.28 ± 0.04**
*Blood pressure and heart rate*		
SBP (mmHg)	122.00 ± 1.79	121.60 ± 1.10
DBP (mmHg)	100.10 ± 1.55	98.40 ± 1.32
MBP (mmHg)	108.80 ± 1.27	109.80 ± 1.23
HR (beats/min)	356.70 ± 1.00	368.90 ± 8.14
*Lipid profile*		
TGs (mg/dl)	68.14 ± 9.96	143.10 ± 23.23*
TC (mg/dl)	63.71 ± 0.94	58.25 ± 3.44

Data are expressed as mean ± SEM, *P < 0.05; ** P < 0.01; ***P < 0.001 versus Control. BW: body weight; DBP: diastolic blood pressure; HR: heart rate; HW: heart weight; LVW: left ventricular weight; MBP: mean blood pressure; SBP: systolic blood pressure; TC: total cholesterol; TGs: triglycerides.

Serum fasting insulin concentration in the HSu-treated rats was significantly elevated when compared with the Control animals (10.83 ± 1.00 *vs* 3.74 ± 1.84 μg/L; p < 0.001) (Figure 1D). A significantly higher value (p < 0.01) of the HOMA-IR index was found in the HSu-treated group, when compared with the Control (Figure 1E). Insulin sensitivity was assessed by the ITT (Figure 1F). The blood glucose levels 120 min. after insulin injection, were significantly elevated in the HSu-treated group than in the Control one (63.86 ± 14.78 *vs* 37.17 ± 7.03 mg/dL; p < 0.01). TRB3 mRNA expression, in the cardiac muscle tissue, was unchanged in both groups (data not shown).

### HSu-diet induces hypertriglyceridemia without obesity and hypertension

Body weight was unchanged between the groups (Table 1). The Hsu-treated rats presented elevated serum triglyceride contents when compared with the Control, while no significant difference was observed in the total-cholesterol concentration between the two groups (Table 1). No significant differences were found in systolic, diastolic or mean blood pressure, as well as in heart rate, between the two groups (Table 1).

### HSu-diet promotes left ventricle hypertrophy with BNP overexpression

**Figure 2:**
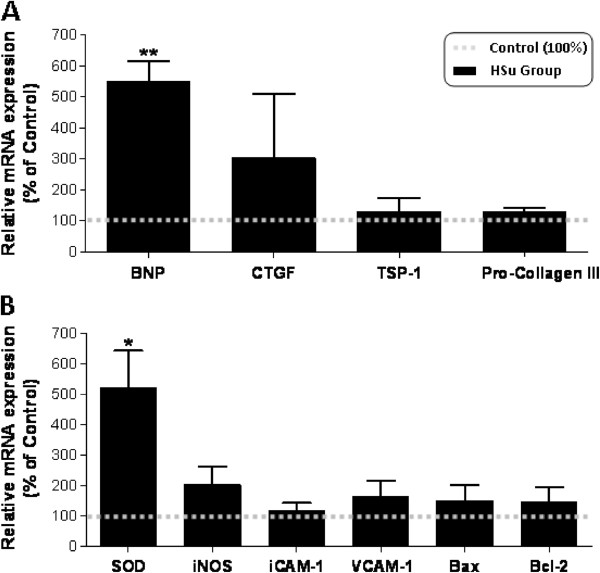
**Cardiac gene expression profile.** mRNA expression of mediators of proliferation, fibrosis and hypertrophy (**A:** BNP, CTGF, TSP-1 and Pro-collagen III) and of endothelial lesion and apoptosis (**B**: SOD, iNOS, ICAM-1, VCAM-1, Bax and Bcl2), after 9 weeks of treatment. (Results are means ± SEM of relative expression in relation to the control; n = 7 per group. *p < 0.05 and **p < 0.01 versus Control group).

**Figure 3:**
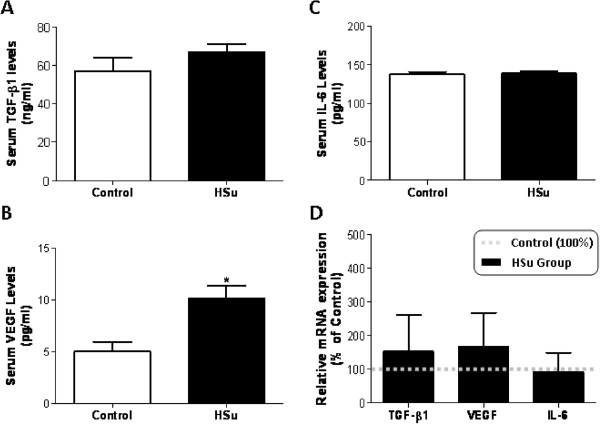
**Serum and cardiac tissue protein/gene expression profile.** Serum TGF-β1 (**A**), VEGF (**B)** and IL-6 (**C**) concentrations and cardiac mRNA expression of TGF-β1, VEGF and IL-6 (**D**), after 9 weeks of treatment. (Results are means ± SEM; relative expression in relation to the control for mRNA levels; n = 7 per group. *p < 0.05 versus Control group).

Absolute and relative left ventricle weight (LVW and LVW/BW) was significantly higher in the HSu-treated rats when compared with the Control animals (p < 0.001 and p < 0.01, respectively) (Table 1). To assess the influence of sucrose consumption on proliferation, hypertrophy and fibrosis, the following mediators were evaluated in serum and/or cardiac tissue: BNP, TGF-β1, TSP-1, pro-collagen III and CTGF. No significant changes were found concerning the cardiac mRNA expression of TGF-β1, TSP-1, pro-collagen III and CTGF, despite a trend to higher values in the HSu animals (Figure 2A). Serum TGF-β1 levels were also identical between groups, despite a trend to higher values in the HSu-treated rats (Figure 3A). However, a significant > 5 fold increase (p < 0.01) in BNP mRNA expression was present in the HSu-treated animals when compared with Control (Figure 2A).

### HSu-diet did not change cardiac markers of apoptosis, inflammation, angiogenesis and endothelial lesions

No changes were found in cardiac muscle for apoptotic markers, Bax and Bcl-2 mRNA expression, between the two groups. iNOS, ICAM-1 and VCAM-1 gene expression was also identical for both groups (Figure 2B). A trend to increased cardiac VEGF mRNA expression was found in the HSu-treated animals, with a significantly higher (p < 0.05) serum VEGF concentration, when compared with the control rats (Figure 3B and D). Both cardiac IL-6 mRNA expression and circulating levels were unchanged between groups, (Figure 3C and D). TNF-α mRNA expression was also identical between groups (data not shown).

### Effect of HSu treatment on redox status markers

**Figure 4:**
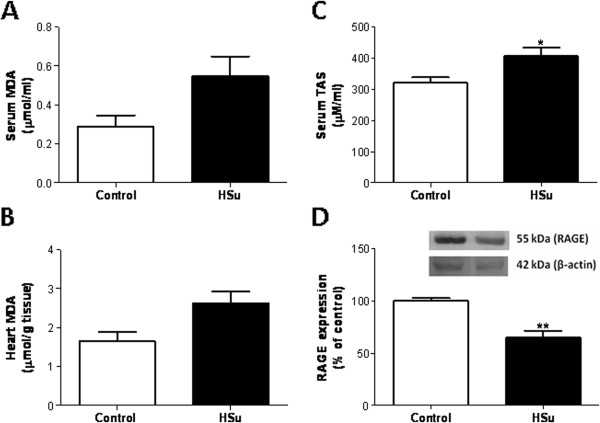
**Serum and cardiac tissue redox status markers.** Serum (**A**) and heart (**B**) lipid peroxidation, serum total antioxidant status (**C**) and cardiac RAGE mRNA expression (**D**), after 9 weeks of treatment. (Results are means ± SEM; relative expression in relation to the control for mRNA levels; n = 7 per group. *p < 0.05 and **p < 0.01 versus Control group).

Both serum and heart tissue lipid peroxidation showed a trend for an increase, although the levels did not achieve statistical significance (Figure 4A and B). However, serum TAS was significantly increased (p < 0.05) in the animals treated with sucrose (Figure 4C), which was accompanied by a significant increase (p < 0.05) in SOD mRNA overexpression and a significant reduction in RAGE levels in cardiac muscle (p < 0.01) (Figure 2B).

## Discussion

### The high-sucrose diet rat as a model of prediabetes

Many reports have demonstrated that consumption of high-sucrose diet induces impaired glucose tolerance and hyperinsulinemia, and decreases insulin sensitivity [27-29]. However, data from models of prediabetes, namely the HSu-treated rat, are scarce [15,16]. Our study demonstrates no differences on glycemia in the fasting state, but a significant increase in the fed (postprandial) state in HSu-treated rats, together with impaired glucose tolerance, hyperinsulinemia and insulin resistance. This is line with previous studies having demonstrated that sucrose consumption produces insulin resistance in rats [19,20], which is characterized by fasting normoglycemia [30,31]. Thus, HSu-evoked impaired glucose tolerance as well as hyperinsulinemia might be triggered by peripheral insulin resistance. In fact, T2DM is recognized as a progressive disorder, which is associated with diminishing pancreatic function over time. However, at an early stage, as it may occur after 9 weeks of high sucrose intake, the presence of insulin resistance must be compensated by hyperinsulinemia to maintain normoglycemia. This is the case in individuals who develop diabetes, a progressive loss of the insulin secretory capacity of β-cells can appear much before the clinical diagnosis [32]. Furthermore, TRB3 (a member of mammalian tribbles homologs protein family), has been recently considered to be a mediator of insulin resistance in DCM [33], explaining our interest in its evaluation in this model. However, our result showed no differences in TRB3 mRNA expression between the groups, suggesting that it might not be impaired in the sucrose-induced prediabetic state.

The present study demonstrated that sucrose intake induced no significant changes on body weight and total cholesterol levels, suggesting that insulin resistance produced in this model is, at least initially, independent of the overt obesity, which is consistent with a previous study [28]. However, elevated serum TGs contents were observed in the HSu-treated rats consistent with other reports [28,34]. This may be a result of *de novo* lipogenesis, the enhanced rate of hepatic TGs synthesis and the decrease in peripheral clearance [35]. In addition, no significant differences in blood pressure and heart rate were observed between HSu-treated rats and the control animals, indicating that 9 weeks of 35% sucrose treatment was unable to cause a significant impact on these parameters. The data is in agreement with a previous work, which failed to show hypertension in high-sucrose diet fed rats [20].

The mechanisms underlying the development of diabetic cardiomyopathy have been widely discussed and seem to involve complex and multifactorial influences. Indeed, as in diabetes, hyperglycemia, hyperinsulinemia and hyperlipidemia might be the initial triggers of significant cellular and molecular changes ultimately leading to cardiac functional and structural impairment, key features of the systolic and diastolic dysfunction associated with diabetic cardiomyopathy. Oxidative stress, endothelial dysfunction, inflammation and apoptosis, are important triggers of cardiac fibrosis and hypertrophy, which are viewed as major structural changes associated with cardiac dysfunction inherent to DCM [15-18]. The existence of earlier cardiac changes in prediabetes has been suggested, but the human studies, and even the animal models, are usually limited because of the coexistence of other perturbations. Our data is consistent with a model of prediabetes/insulin resistance, characterized by fasting normoglycemia, impaired glucose tolerance, hyperinsulinemia, insulin resistance, together with hypertriglyceridemia, important features to regarding their direct impact on cardiac tissue, independently of other confounding factors, such as hypertension and obesity.

### Early markers of cardiac hypertrophy and/or fibrosis: focus on BNP overexpression

Cardiomyocyte hypertrophy, as well as subsequent apoptosis and myocardial fibrosis, are structural hallmarks of DCM [36,37]. Previous studies have shown that cardiomyocyte hypertrophy in diabetes is regulated at the transcriptional level [38,39]. Thus, it is important to examine molecular biomarkers of the putative cardiac alterations. In this study, we evaluated cardiac mRNA levels of TGF-β1, TSP-1, pro-collagen III, CTGF and BNP, which have been associated with cardiac hypertrophy and/or fibrosis. Indeed, TGF-β1 plays a role in cell growth, differentiation, apoptosis, inflammatory processes and gene expression [40] and has consistent implications in DCM due to pro-fibrotic properties, including the expression of many matrix proteins, such as collagens. In the hearts of diabetic patients, collagen remodeling occurs in the perimysium and perivascular region, mainly as a result of an increased accumulation of type III collagen [41], which has been related to left-ventricular diastolic and systolic dysfunction in DCM [42,43]. Additionally, TSP-1 is an adhesive glycoprotein that influences cellular phenotype and the structure of the extracellular matrix [44]. It has been reported that high glucose levels stimulates TGF-β1 bioactivation by fibroblasts through up-regulation of TSP-1 [45]. CTGF is another potent pro-fibrotic protein that plays important roles in tissue and organ fibrosis and it is increasingly being implicated in structural and functional abnormalities in the diabetic heart [46,47]. In addition, BNP, a peptide hormone released from the cardiac ventricles in response to pressure and volume overload, is among the most relevant molecular markers of cardiac hypertrophy [48]. In our study, no significant differences were found in cardiac gene expression of TGF-β1, pro-collagen III, TSP-1 and CTGF between the groups, despite a trend to increased values in the HSu animals for all mediators. However, BNP gene expression was significantly higher in animals under sucrose treatment, which is in agreement with the LV hypertrophy, viewed by the relative increase in LV weight. Thus, collectively, this data indicate that a high sucrose diet can promote cardiac trophism in a prediabetic state, with BNP the earlier biomarker of those alterations, in agreement with a recent study suggesting BNP as a useful marker for screening preclinical ventricular diastolic dysfunction in patients prone to develop cardiovascular complications, such as uncontrolled diabetics [49]. Cardiac hypertrophy and fibrosis are usually associated with apoptosis [50]. In this study, no significant changes in Bax and Bcl-2 cardiac gene expression were found. Whether these are or not related with the anti-apoptotic effect of insulin in the absence of hyperglycemia, deserves further evaluation [51].

### Markers of endothelial dysfunction and angiogenesis

Endothelial dysfunction is one of the mechanisms involved in the pathogenesis of DCM. Inducible NOS is an enzyme with recognized implications in ROS production, particularly under inflammatory conditions, and it is also involved in insulin resistance [52]. Activation of iNOS contributes to the pathogenesis of CVD, probably because of excessive release of NO and generation of ROS. Ropelle et al. (2013) recently demonstrated that iNOS overexpression induced by aging is associated with increased S-nitrosation of major proteins involved in insulin signalling, thus reducing insulin sensitivity in skeletal muscle, which is prevented in aged iNOS-null mice [53]. In addition, iNOS deficiency protects from high-fat diet-induced insulin resistance in the obese Zucker rat model [54]. In our model, no significant changes in cardiac iNOS gene levels were found, despite a trend for higher values in the insulin resistant animals. Lee et al. (2009) suggest that upregulation of iNOS may be a protective mechanism against excessive contraction, abnormal signalling resulting from oxidative stress and enhanced inflammation in the diabetic vasculature [55]. Whether this hypothetic protection is present or not in our model, needs additional investigation. Endothelial dysfunction was also assessed by the expression of adhesion molecules, such as ICAM-1 and VCAM-1, which have been associated with the development of CVD in diabetic patients [56,57]. In our model, however, no differences in cardiac ICAM-1 and VCAM-1 gene expression were found, suggesting they could be later markers of these changes. Another mediator of vascular changes found in diabetic patients is VEGF, promoting angiogenesis and increasing vascular permeability [58,59]. We observed a trend for increased levels in cardiac tissue VEGF in HSu-treated rats, which was accompanied by increased serum protein levels, suggesting its direct involvement.

### Markers of inflammation and redox status

Elevated inflammatory cytokines have been found in circulation and in the diabetic hearts of T2DM patients, contributing to heart failure [60-62]. Cardiac overexpression of TNF-α has been associated with cardiac hypertrophy and fibrosis, as well with left ventricular dysfunction [63,64] and IL-6 has been also described as an inducer of myocardial damage by promoting LV dysfunction and cardiac hypertrophy under acute myocardial infarction [65]. In our study, however, no significant changes were found in either the serum or cardiac levels for IL-6 and cardiac TNF-α, suggesting that inflammation is not the key factor of the early changes related to cardiomyopathy.

In diabetes, oxidative stress has been recognized as an important player in the mechanism underlying DCM. However, in a state of insulin resistance without hyperglycemia, as it occurs in the early stages of diabetes (prediabetes), the contribution of ROS and consequent oxidative stress to the heart remains to be elucidated. In our model, the absence of markers of increased inflammation was accompanied by slight changes in the redox status measured. We found a trend for increased MDA levels (a marker of lipid peroxidation) in both the serum and heart tissue of HSu-treated rats. However, a significantly increased in serum TAS (a measure of total antioxidant capacity) was observed in the HSu rats, accompanied by an overexpression of the cardiac SOD gene, as well as a reduced expression of RAGE. These might collectively be viewed as compensatory actions against the putative increase in ROS. Furthermore, our model presented hyperinsulinemia, which was still able to normalize blood glucose levels, despite the impaired glucose tolerance. Thus, the putative generation of ROS, as suggested by the small increase in serum and heart lipid peroxidation, seems to be compensated by additional generation of antioxidants, observed by the significantly increased serum TAS and cardiac gene expression of SOD. Furthermore, the observed under-expression of RAGE in cardiac tissue might be also viewed as a compensatory mechanism, protecting the cardiac tissue. Indeed, we cannot exclude the possibility that longer exposure times of sucrose may lead to reduction of these antioxidant defence mechanisms and, consequently, to toxic effects induced by the oxidative stress aggravating cardiac function.

Hyperglycemia has been indicated as one of the major players in DCM, due to activation of several mechanisms, including promotion of oxidative stress. In our model, the modifications present at an early stage (hyperinsulinemia, insulin resistance, fasting normoglycemia) before over diabetes (defined as prediabetes), might be eventually linked with the impact of impaired glucose tolerance (fed, or postprandial, hyperglycemia). Additional studies are needed to better understand the underlying molecular and cellular mechanisms responsible for the effects of HSu in prediabetes, as well as to elucidate whether hyperglycemia is, or not, the major driving force for the overt cardiac changes underlying cardiomyopathy.

## Conclusion

This animal model of prediabetes/insulin resistance, induced by high sucrose consumption during 9 weeks, could be an important tool to evaluate the cardiac impact of dysmetabolism (mainly hyperinsulinemia and impaired glucose tolerance, with fasting normoglycemia), without confounding factors such as obesity and hypertension. Left ventricle hypertrophy is the main characteristic and BNP overexpression seems to be the best early marker.

## Abbreviations

BNP: Brain natriuretic peptide; BSA: Bovine serum albumin; BW: Body weight; CAD: Chronic artery disease; CTGF: Connective tissue growth factor; CVD: Cardiovascular diseases; DBP: Diastolic blood pressure; DCM: Diabetic cardiomyopathy; DM: Diabetes mellitus; GTT: Glucose tolerance test; HF: Heart failure; HOMA-IR: Homeostasis model assessment-Insulin Resistance; HR: Heart rate; HSu: High sucrose; HW: Heart weight; ICAM-1: Intercellular adhesion molecule-1; IFG: Impaired fasting glucose; IGT: Impaired glucose tolerance; IL-6: Interleukin-6; iNOS: Inducible nitric oxide synthase; ITT: Insulin tolerance test; FPG: Fasting plasma glucose; FRAP: Ferric reducing antioxidant potential; LV: Left ventricle; LVW: Left ventricle weight; MDA: Malondialdehyde; MBP: Mean blood pressure; RAGE: Receptor of advanced glycation endproducts; SBP: Systolic blood pressure; SOD: Superoxide dismutase; TAS: Total antioxidant status; TBARs: Thiobarbituric acid reactive-species, TC, Total cholesterol; TGF-β1: Transforming growth factor β-1; TGs: Triglycerides; TNF-α: Tumour necrosis factor alpha; TRB3: Tribbles 3; TSP-1: Thrombospondin-1; VCAM-1: Vascular cell adhesion molecule-1; VEGF: Vascular endothelial growth factor.

## Competing interest

The authors report no competing interest.

## Authors' contributions

SN, EC, FCP and FR conceived and designed the study protocol. SN, ES, JF, SV and EC performed the analytical assays. SN, EC, FCP and FR prepared the manuscript. All authors have read and approved the manuscript.
